# Tightening slip knots in raw and degummed silk to increase toughness without losing strength

**DOI:** 10.1038/srep18222

**Published:** 2016-02-12

**Authors:** Maria F. Pantano, Alice Berardo, Nicola M. Pugno

**Affiliations:** 1 Laboratory of Bio-inspired & Graphene Nanomechanics, Department of Civil, Environmental and Mechanical Engineering, University of Trento, Via Mesiano 77, 38123 Trento, Italy; 2 Center for Materials and Microsystems, Fondazione Bruno Kessler, Via Sommarive 18, 38123 Povo (TN), Italy; 3 School of Engineering and Materials Science, Queen Mary University of London, Mile End Road, London E1 4NS, U.K

## Abstract

Knots are fascinating topological elements, which can be found in both natural and artificial systems. While in most of the cases, knots cannot be loosened without breaking the strand where they are tightened, herein, attention is focused on slip or running knots, which on the contrary can be unfastened without compromising the structural integrity of their hosting material. Two different topologies are considered, involving opposite unfastening mechanisms, and their influence on the mechanical properties of natural fibers, as silkworm silk raw and degummed single fibers, is investigated and quantified. Slip knots with optimized shape and size result in a significant enhancement of fibers energy dissipation capability, up to 300–400%, without affecting their load bearing capacity.

Knots are intriguing topological elements, with a variety of examples appearing in fine arts (Fig. 1a) as well as many scientific fields, including mathematics^1^, polymer science^2^,^3^, colloids^4^,^5^, fluids^6^, chemistry^7^,^8^, biology^9^, and obviously engineering^10^. Knots can be introduced by human hand^11^, but many biological systems, like proteins and DNA, naturally form knotted configurations^12^, with their function being still mysterious and under debate^13^. Herein, we investigate how the presence of knots is able to affect the mechanical properties of natural fibers, as silkworm silk. Indeed, it has been recently proposed that knots can significantly improve the energy dissipation capability (i.e., toughness) of materials^10^.

Silkworm silk has been implemented for centuries in textile and medical industries, with recent application in composites^16^, tissue engineering scaffolds^17^,^18^ and drug delivery^19^, and is now receiving a renewed interest, as natural materials can address the need for sustainable and biodegradable structural components^20^.

Thus, we exploit potential knotted structures to artificially increase the toughness of silkworm silk without any genetic modification or chemical treatment, but reproducing at the microscale the same toughening function which sacrificial bonds have in highly coiled macromolecules^14^,^15^. In fact, as the breakage of weak bonds (i.e., sacrificial bonds) reveals a hidden length in macromolecules, which can thus be further stretched without breaking their backbones, the knots release in our samples provide additional length to silk fibers, which can thus be further elongated before failure.

From a mechanical point of view, silk fibers extracted from silkworm cocoons have been reported with remarkable mechanical properties, i.e., Young modulus up to 16 GPa^21^, fracture strength up to 600 MPa^22^ and toughness of 6·10^4^ J/kg^23^, even though these cannot compete with those characterizing spider silk dragline^24^, having fracture strength of 1.3 GPa and toughness of 16·10^4^ J/kg^23^. However, since spiders offer a significantly smaller yield capability, which hinders their silk to be fully implemented in a massive industrial production^25^, it would be desirable to combine the advantages offered by both such biomaterials, thus developing methods to provide silkworm silk with spider silk performances. Apart from genetic modification and chemical treatment^26^,^27^, mechanical properties of silkworm silk were showed to be improvable by artificially increasing the reeling speed of silk from the silkworm^23^.

In the present paper, we focus on a knot-based strategy^10^ to improve the toughness of as-produced silkworm silk. Our strategy^10^ requires the introduction within single silk fibers of a sliding frictional element, namely a knot with a proper topology and optimized shape and size.

While knots typically encountered in biological or chemically synthetized molecular systems cannot be loosened without breaking (chemically or mechanically) the strand where they are tightened, with only rare exceptions^28^, the knots introduced in our fibers were designed as able to unfasten as their opposite ends are pulled apart.

In fact, this is a necessary condition to fully exploit the knot friction potential and avoid any stress concentration, which can trigger premature failure of the fiber, thus compromising its load bearing capacity.

Hereby, in the present study attention was focused on slip or running knots. In particular, two different topologies involving opposite unfastening mechanisms were implemented and optimized in case of single silk fibers (Fig. 1b,c). Furthermore, since silk extracted directly from cocoons usually undergoes a degumming process before being processed in industrial applications, in our knot optimization we considered both natural (i.e., extracted directly from a cocoon) and degummed (i.e., extracted from degummed cocoons) fibers, in order to capture potential differences due to the different surface friction coefficients. Then, tensile tests were performed on both knotted and unknotted control samples in order to evaluate the toughness enhancement due to the knot presence.

## Results

**Figure 2:**
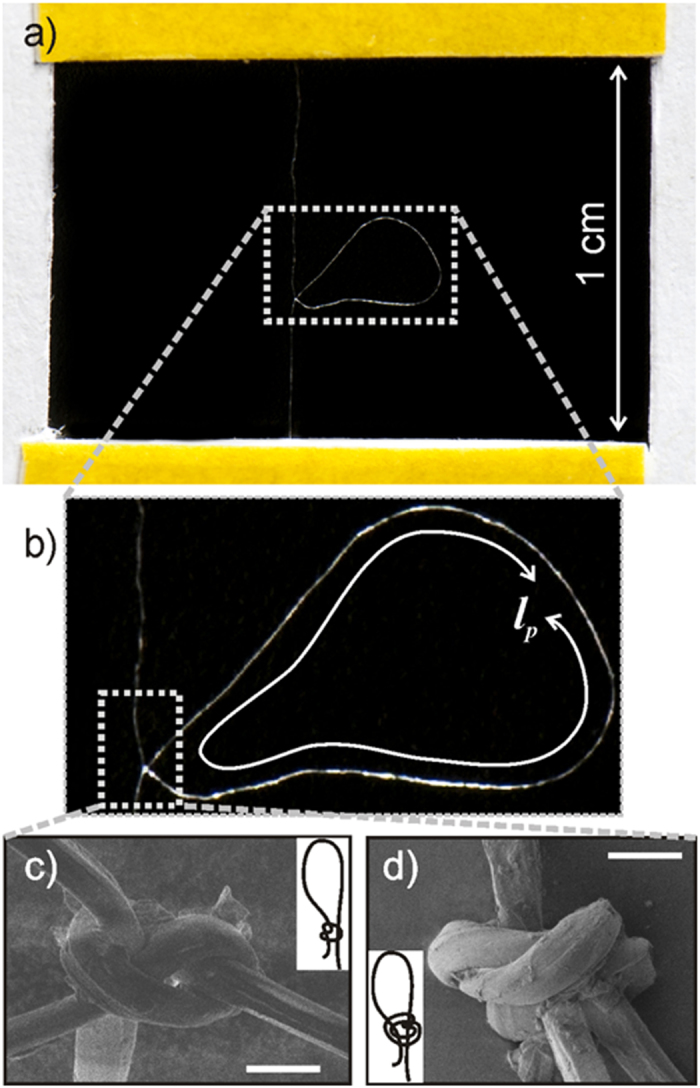
(**a**) A degummed silk fiber, provided with an optimized knot, spanning over a paper frame prepared for nanotensile testing. The knot, either single (STSK) or double (DTSK) turned slip knot, is characterized by two main parameters, the loop length, *l*_*p*_, and the knot diameter, as shown in the zoomed view (**b**). SEM images of the single (**c**) and double (**d**) turned slip knot. Scale bars: 20 μm.

In the present experiments, we compared the effectiveness of two kinds of slip (or running) knots, where the fiber was turned either once (single turned slip knot, STSK, also known as *noose*) or twice (double turned slip knot, DTSK, also known as *overhand loop*) at the bottom of a loop (Fig. 2). In both cases, the fiber is allowed to slide throughout the knot, in order to promote energy dissipation, but undergoes a different unfastening mechanism. In fact, while the first kind of knot is always able to unfasten, even when extremely tight, as it loosens when the fiber ends are pulled apart, the second one poses much more issues, since, on the contrary, it becomes tighter as the fiber is pulled. For both untreated and degummed silk, either knot topologies were optimized in order to fulfill two main requirements. First, the knot has to be sufficiently tight in order to extend the strain interval where the fiber experiences a relatively high stress. Second, this must be able to unfasten as the fiber opposite ends are pulled apart, in order to not affect the fiber fracture strength.

**Figure 3:**
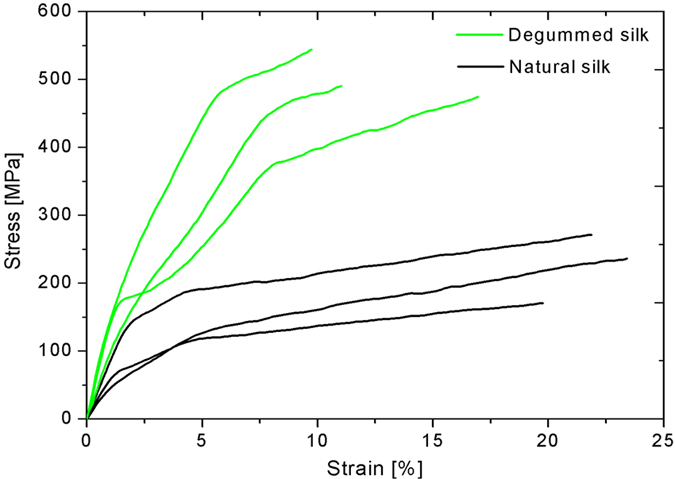
Stress-strain curves derived from tensile tests carried out on single untreated baves (black line) and degummed fibers (green line), both showing significant variability.

Reference values of silk toughness were derived from tensile testing of control untreated baves and degummed single silk fibers with no knot implemented (i.e., toughness is proportional to the area under sample stress-strain curve) (Fig. 3). Then, in order to evaluate the toughness increase due to the knot introduction, we performed a wide experimental campaign, with the corresponding results reported in the Supplementary Information.

However, extracting meaningful data from tensile tests on silk is not straightforward. In fact, as expected from the literature, the stress-strain curves of control silk fibers showed significant variability (Fig. 3), which causes in turn variability in terms of mechanical properties, included toughness. Such variability is mainly caused by fluctuations in the fiber diameter, which is in turn dependent of many factors closely related to the silkworm nature^29^, such as mode and speed of the spinning process. Furthermore, fiber diameter can not only vary in size^20^,^22^ but also in shape over the same cocoon^21^. However, as common practice in the literature^21^, we considered the fibers as provided with a circular cross-section.

The diameter of each tested fiber was evaluated from observation under either optical or scanning electron (SEM) microscope, providing average values of 21 μm and 12 μm for natural and degummed fibers, respectively.

**Figure 4:**
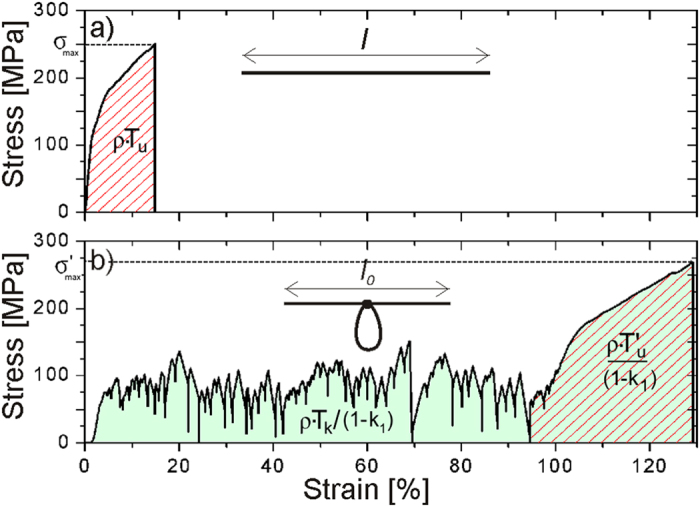
(**a**) Stress-strain curve of an unknotted natural fiber with length l and toughness modulus *T_u_* (equal to the marked area divided by the fiber density *ρ*). (**b**) Stress-strain curve of a knotted natural fiber with length *l*, toughness modulus *T_k_* (equal to the shadowed area multiplied by (1-*k1*)/*ρ*, where *k1*=1-*l0/l*), which was extracted from a cocoon region adjacent to the unknotted fiber (**a**). The presence of the knot modifies the shape of the stress-strain curve (**a**), introducing a plastic-like plateau and leaving the final region (marked with lines and equal to *T_u_*’, the fiber toughness modulus after knot release, multiplied by *ρ*/(1-*k1*)) almost corresponding to the stress-strain curve of the same fiber with unknotted configuration. The strain interval within this final region appears larger than in (**a**) since it is computed with respect to *l0* instead of *l*.

For a fiber without any knot, the energy dissipated per unit mass, *T*_*u*_, e.g., toughness modulus, can be computed from its stress-strain curve as (Fig. 4a):


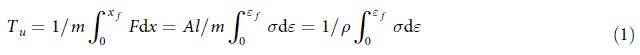


where *m* is the fiber mass, *x*_*f*_ is the displacement at fracture, *F* is the applied force, *A* is the fiber cross sectional area, *l* is the fiber initial length, *ρ* is the volumetric density, 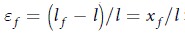 is the fracture strain, *l*_*f*_ is the fiber final length, and 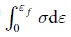 is the area under the stress-strain curve. Such expression has to be slightly adjusted if knotted fibers are instead considered. In fact, if a knotted fiber with still length *l* is tested (Fig. 4b), its toughness modulus can be computed as:


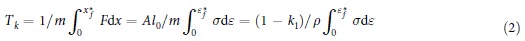


where 
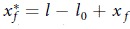
, *l*_*0*_ is the initial length equal to the distance between the fiber opposite ends (Fig. 4b), 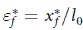, and 
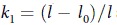
 accounts for the difference between *l*_*0*_ and *l*^10^.

In order to derive quantitative results of knot induced toughness increase, which is not affected by variability of silk mechanical properties, we pursued the following strategy when comparing the toughness of a knotted sample computed according to Eq. (2) with the toughness of a control sample calculated according to Eq. (1). In fact, when possible, we referred toughness comparison to the same fiber; alternatively, as reference value we considered the toughness of an unknotted fiber which was extracted from a cocoon region adjacent to that of the knotted fiber, thus expecting a minimal variation in their physical and mechanical properties.

In fact, in some cases, after a series of loading and unloading events due to knot fastening and unfastening, the knot loosens completely, leaving the stress-strain curve of knotted fibers collapsing to the stress-strain curve of the corresponding unknotted samples, as shown in Fig. 4. This indicates that the mechanical behavior of silk is not affected by loading-unloading cycles, confirming previous results derived from dynamic tests^22^ and allowing the final part of the curve (highlighted in Fig. 4b) to be considered as the stress-strain curve related to the unknotted configuration of the same fiber. In such situation, the ratio between the toughness of the knotted fiber, *T*_*k*_, and the toughness of the corresponding unknotted fiber, *T*_*u*_’, was computed as:


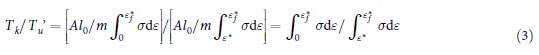


where 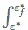 is the area under the final part of the stress-strain curve, where the knot is completely released.

**Figure 5:**
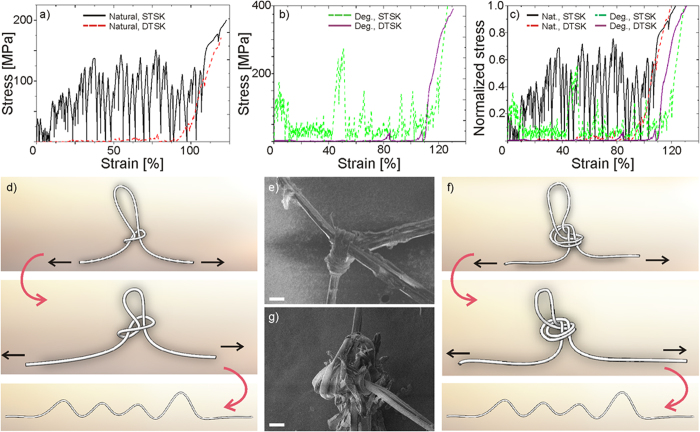
Stress-strain curves of natural (a,b) degummed silk fibers with optimized single or double turned slip knot. (**c**) Comparison between the normalized stress-strain curves obtained for natural and degummed single silk fibers provided with optimized knots. Stress values are normalized with respect to fracture strength. (**d**) Unfastening mechanism of the single turned slip knot, which tends to loosen as the fiber ends are pulled apart. Such knot can always be released, even when extremely tight, as shown in the SEM image (**e**). (**f**) Unfastening mechanism of the double turned slip knot, which tends to further tie as the fiber ends are pulled apart. Thus, if this knot is too tight at the beginning of the test, it cannot be released, as occurred in the natural silk fiber reported in the SEM image (**g**) which broke at the knot entrance. The sericin coating looks significantly damaged by friction. Scale bars: 30 μm.

In other tests, the stress-strain curve of knotted fibers showed a well-defined plateau up to the end (as the curve corresponding to a natural fiber provided with single turned slip knot reported in Fig. 5a). Hereby, it is not possible to identify the final region of the stress-strain curve as the stress-strain curve corresponding to a plain sample. Then, we derived a reference toughness value from testing an unknotted fiber initially adjacent to the fiber where knot was then implemented.

Thus, in order to compare toughness values of a knotted and corresponding unknotted fiber, the area under the stress-strain curve of the knotted fiber has to be scaled by the factor (1–*k*_*1*_):


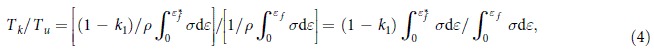


where the symbols have the same meaning as before. Results obtained for both Tu’ and Tu are reported in Table 1.

In the presented analysis, the toughness increase was evaluated according to expression (3) for degummed fibers provided with either single or double turned slip knot and natural fibers provided with double turned slip knot. Expression (4) was used instead in most of the cases to evaluate the toughness increase in natural fibers with single turned slip knot.

Figure 5a,b reports example stress-strain curves derived for natural and degummed single silk fibers with optimized single or double turned slip knot.

With respect to unknotted control samples (Fig. 3), many differences emerge. First, as expected, the knot presence extends the strain interval (i.e., fibers provided with a knot reach a bigger apparent strain) and introduces an artificial plateau, characterized by a series of peaks and drops, corresponding to partial fastening and unfastening of the fiber in the knot and related stick-slips. In particular, a well-defined plastic-like plateau appears especially when the single turned slip knot topology is considered and this is more evident for natural fibers than for degummed fibers. This means that natural fibers with this knot topology can be constantly high stressed throughout the whole test, causing energy dissipation to be strongly enhanced. Such observations are quantitatively confirmed by values reported in Table 1.

In fact, the single turned slip knot topology allowed to significantly enhance toughness of both natural and degummed fibers, with more than 350% and 250% increase in the optimal configuration, respectively. On the contrary, the double turned slip knot topology resulted to be sensibly less performing, with comparable toughness increase around 110% for both natural and degummed fibers.

## Discussion

The results shown in the previous section can be explained looking at the unfastening mechanism involved in either knot topology. In fact, the single turned slip knot tends to loosen during the test (Fig. 5d). Hereby, it is possible to start from a very tight configuration (Fig. 5e), which provides the fiber to be significantly stressed throughout the whole test within a relatively wide apparent strain interval, which allows to more than quadrupling toughness (see Supplementary Information). On the contrary, the double turned slip knot tends to further tie as the fiber is pulled (Fig. 5f). Thus, in order to release completely the fiber without any damage, it is necessary to start from a very loose configuration. This, however, causes the fiber not to be very stressed, except at the end of the test, providing a much less significant toughness enhancement.

On average, with reference to the single turned slip knot, higher toughness values were reported for natural silk than for degummed silk. This is related to the possibility for natural fibers to dissipate more energy by friction, thus reaching a stress plateau much closer to their fracture strength, as it emerges if the stress values reported in Fig. 5a,b are normalized with respect to the corresponding fracture strength (Fig. 5c). The double turned slip knot topology provided instead comparable results for both natural and degummed fibers.

Such different behavior can be explained considering the role played by sericin coating. In fact, due to sericin, natural silk fibers are less smooth than degummed fibers, thus being more prone to friction as they run through the knot. However, when the knot is always able to unfasten (e.g., STSK), this is an added value and contributes favorably to further increase the fiber toughness. On the other side, when it is difficult for the fiber to run throughout its loop as the knot tends to tie during tensile tests (e.g., DTSK), any additional friction source can further hinder sliding, causing damage and premature failure of the fiber (Fig. 5e–g). Thus, it is necessary to start from a very loose configuration, which minimizes or even cancels out the beneficial effect of sericin on friction enhancement.

## Conclusions

In summary, we have presented the effect of slip knots on the toughness of single silkworm silk fibers applying the strategy proposed in ref [10]. Our study demonstrates that, under optimized conditions, a slip knot introduced within the fiber can increase its energy dissipation capability, without causing significant damage to it and avoiding significant stress concentration at the knot entrance. Here, two different topologies were considered, with the fiber turned either once or twice at the bottom of a loop. While both topologies allow the fiber to slide within their loop, thus promoting energy dissipation, they involve a different unfastening mechanism, with the knot prone to either untie or tie, as the fiber ends are pulled apart. The first topology with the fiber turned once at the bottom of a loop provided the best results, with more than three times toughness enhancement compared to a reference unknotted sample.

We believe that the silk toughness could be further increased by considering longer loop to fiber length ratio than that of our experiments, or introducing multiple slip knots within the same fiber. Thus, the results presented in our work should serve as a guide for future investigation of more complex knots, like those implemented in textile industry, in order to provide new tools for optimizing systems where energy dissipation is highly requested.

## Methods

### Sample preparation

For the experiments presented in the present paper, single silk fibers were extracted from untreated and degummed cocoons of domestic *Bombyx mori* silkworm. Some of the isolated fibers were manipulated by tweezers in order to introduce a knot, while the others were left plain and used as control samples. From a structural point of view, natural silk fibers (baves) are composed of two filaments (known in the literature as brins), mainly consisting of fibroin, which are coated with a sericin layer binding them together. Since sericin does not contribute to load bearing capacity of the bave^30^, this was removed through a typical degumming process^31^, thus allowing to obtain bare fibroin fibers separated one from another. The process implemented in the present experiments followed a typical procedure^31^, consisting of boiling twice the cocoon with 1.1 g/L and 0.4 g/L Na_2_CO_3_ (anhydrous, minimum 99%, from Sigma Aldrich) water solution for one hour each time. This allowed to remove any sericin traces, obtaining bare fibroin fibers, which were then washed against distilled water and air-dried.

Some samples were left plain and used as control samples, while others were provided with either single or double turned slip knots. Starting from a fiber length (*l*) of 20 mm and a distance between the fiber ends (*l*_*0*_) of 10 mm, the optimal single turned slip knot geometry which allowed to maximize the fiber energy dissipation capability had a very small knot diameter with a loop length (*l*_*p*_) of about 10 mm (Fig. 2). In fact, as this kind of knot tends to loosen during tensile tests, it is convenient to start from the tightest possible configuration. On the contrary, it was not possible to perform successful experiments with a fiber length of 20 mm and *l*_*0*_equal to 10 mm, provided with double turned slip knot. In fact, knots with this size could not completely unfasten during tensile tests. Thus, an optimization process was carried out in order to guarantee the knot to completely release during a test on a fiber with the longest possible loop (for dissipating the highest possible energy), still keeping *l*_*0*_ = 10 mm. This had the following geometry: knot diameter of 6 ± 0.3 mm (with about 12 mm of fiber involved within the knot), and loop length (*l*_*p*_) of 6 mm.

### Tensile tests

Both untreated baves and degummed single silk fibers were tested at room temperature through a nanotensile testing machine (Agilent T150 UTM) and at a strain rate of 0.001 s^−1^ in case of control samples and 0.002 s^−1^ in case of samples provided with knots.

## Additional Information

**How to cite this article**: Pantano, M. F. *et al*. Tightening slip knots in raw and degummed silk to increase toughness without losing strength. *Sci. Rep.* **6**, 18222; doi: 10.1038/srep18222 (2016).

## Supplementary Material

Supplementary Information

## Figures and Tables

**Figure 1:**
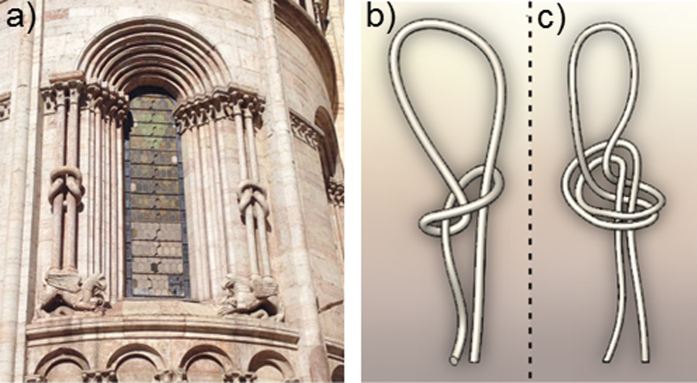
(**a**) Duomo of Trento (Italy): detail of the apse loggia with a couple of knotted columns (XIII century). *Photograph by A.B.* Schematics of the knots designed in our experiments on single silk fibers: the single turned slip knot, STSK, (**b**) and the double turned slip knot, DTSK, (**c**) where the fiber is turned either once or twice at the bottom of a loop.

**Table 1:** 

	Diameter [µm]	Strength of unknotted fibers [MPa]	Toughness modulus of reference unknotted fiber, T_u_ [J/g]	Knot topology	Strength [MPa]	Toughness modulus, T_k_ [J/g]	Toughness modulus after unfastening, Tu’ [J/g]	Friction stress/strength
Raw silk	21 ± 2	219 ± 68	20 ± 11	STSK	229 ± 50	45 ± 12	15 ± 9	> 8 %
					216 ± 46	19 ± 8	16 ± 7	< 8 %
				DTSK	–	–	–	> 8 %
					237 ± 53	17 ± 18	16 ± 8	< 8 %
Degummed silk	12 ± 2	502 ± 141	28 ± 12	STSK	343 ± 104	28 ± 9	11 ± 7	> 8 %
					463 ± 120	36 ± 18	28 ± 19	< 8 %
				DTSK	–	–	–	> 8 %
					434 ± 146	29 ± 17	27 ± 17	< 8 %

Strength or toughness (Tu) of control unknotted fibers. Toughness modulus (Tk), strength or toughness modulus after unfastening (Tu’) of knotted fibers with single turned slip knot (STSK) or double turned slip knot (DTSK) topologies. For each knot topology, two sets of data are provided, corresponding to samples with average stress in the strain interval 0% - 40% of their strain at break (i.e., friction stress) above or below the 8% of their strength. Such threshold value was considered as the minimum friction stress required for knots to be efficiently implemented.
