# Supplementary material for: Tightening slip knots in raw and degummed silk to increase toughness without losing strength
Source: Sci Rep. 2016 Feb 12;6:18222. doi: 10.1038/srep18222 (PMC4751510; doi:10.1038/srep18222)
Supplement: Supplementary Information [file srep18222-s1.docx]

**Supplementary Information**

Supplementary Tables S1-S4 report the results obtained from tensile tests on both raw and degummed single silk fibers provided with Single Turned Slip Knot (STSK) and Double Turned Slip Knot (DTSK) topologies. In particular, a variety of data can be found, including the strength and the toughness modulus (T_k_) of knotted fibers, the toughness modulus of knotted samples computed after complete knot release (T_u_’), the toughness modulus of a reference unknotted fiber (T_u_) when T_u_’ could not be clearly identified. Toughness modulus values were computed considering a density of 1.4 g/cm^3^ [S1]. Supplementary Tables S1-S4 include also the values of the mean stress reached by samples over 0% - 40% of their strain at break. In fact, the average stress value reached by knotted fibers at the beginning of tensile tests is indicative of the friction force and thus of the tightness quality of implemented knot. The values of toughness modulus reported in the first or fifth row of Table 1 of the main text are computed as average over those values corresponding to samples which reached an average friction stress above the threshold value of 8% of the sample strength. Samples satisfying such requirement were highlighted in both Supplementary Table S1 and S3. On the contrary, the values of toughness modulus reported in the second or sixth row of Table 1 of the main text are computed as average over those values corresponding to samples which reached an average friction stress below the threshold. In case of DTSK, neither sample reached a friction mean stress above the threshold, thus demonstrating its low efficiency if compared to STSK topology.

Supplementary Table S1. Raw silk fibers with single turned slip knot: Strength, average friction stress/strength over 0% - 40% of the strain at break, toughness modulus (T_k_), toughness modulus after knot unfastening of knotted fibers (T_u_’), toughness modulus of reference unknotted samples (T_u_).

| Material | Knot topology | Sample number | Strength [MPa] | Friction stress/strength | Toughness modulus, T_k_ [J/g] | Toughness modulus after unfastening, T_u_' [J/g] | Toughness modulus, T_u_ [J/g] |
| --- | --- | --- | --- | --- | --- | --- | --- |
| Raw silk | STSK | 1 | - | - | - | - | - |
| Raw silk | STSK | 2 | 305 | 25.5% | 67.7 | - | 32.6 |
| Raw silk | STSK | 3 | 246 | 29.0% | 53.1 | - | 19.6 |
| Raw silk | STSK | 4 | 269 | 25.0% | 52.1 | 23.1 |  |
| Raw silk | STSK | 5 | 200 | 23.1% | 37.1 | - | 8.5 |
| Raw silk | STSK | 6 | 196 | 38.6% | 42.5 | - | 8.5 |
| Raw silk | STSK | 7 | 115 | 0.4% | 3.5 | 2.6 | - |
| Raw silk | STSK | 8 | 226 | 1.0% | 18.8 | 17.4 | - |
| Raw silk | STSK | 9 | 191 | 1.3% | 22.1 | 20.4 | - |
| Raw silk | STSK | 10 | 220 | 6.2% | 20.3 | 14.2 | - |
| Raw silk | STSK | 11 | 246 | 3.1% | 25.1 | 21.8 | - |
| Raw silk | STSK | 12 | 183 | 30.2% | 39.4 | 7.7 | - |
| Raw silk | STSK | 13 | 165 | 24.7% | 27.8 | 7.3 | - |
| Raw silk | STSK | 14 | - | - | - | - | - |
| Raw silk | STSK | 15 | 269 | 5.1% | 30.7 | 23.5 | - |
| Raw silk | STSK | 16 | 244 | 1.4% | 13.6 | 11.9 | - |
| Raw silk | STSK | 17 | 242 | 4.1% | 21.5 | 17.6 | - |
| Raw silk | STSK | 18 | 268 | 17.0% | 41.7 | 22.9 | - |
| Raw silk | STSK | 19 | 191 | 6.8% | 18.0 | - | 8.5 |

Supplementary Table S2. Raw silk fibers with double turned slip knot: Strength, average friction stress/strength over 0% - 40% of the strain at break, toughness modulus (T_k_), toughness modulus after knot unfastening of knotted fibers (T_u_’).

| Material | Knot topology | Sample number | Strength [MPa] | Friction stress/strength | Toughness modulus, T_k_ [J/g] | Toughness modulus after unfastening, T_u_' [J/g] |
| --- | --- | --- | --- | --- | --- | --- |
| Raw silk | DTSK | 1 | 235 | 0.1% | - | - |
| Raw silk | DTSK | 2 | 297 | 0.0% | 14.1 | 13.9 |
| Raw silk | DTSK | 3 | 312 | 0.5% | 31.6 | 31.2 |
| Raw silk | DTSK | 4 | 171 | 0.1% | 8.0 | 7.3 |
| Raw silk | DTSK | 5 | 243 | 0.5% | 18.7 | 18.3 |
| Raw silk | DTSK | 6 | 175 | 0.6% | 12.4 | 10.6 |
| Raw silk | DTSK | 7 | - | - | - | - |
| Raw silk | DTSK | 8 | 111 | 0.8% | - | - |
| Raw silk | DTSK | 9 | 117 | 0.8% | - | - |
| Raw silk | DTSK | 10 | 198 | 1.8% | - | - |
| Raw silk | DTSK | 11 | 157 | 1.1% | - | - |
| Raw silk | DTSK | 12 | 7 | - | - | - |
| Raw silk | DTSK | 13 | 236 | 0.9% | - | - |
| Raw silk | DTSK | 14 | 226 | 0.5% | - | - |
| Raw silk | DTSK | 15 | 212 | 0.5% | 11.9 | 10.5 |
| Raw silk | DTSK | 16 | 219 | 0.4% | 12.5 | 11.7 |
| Raw silk | DTSK | 17 | 267 | 0.2% | - | - |
| Raw silk | DTSK | 18 | 209 | 0.4% | - | - |
| Raw silk | DTSK | 19 | 273 | 0.4% | 26.1 | 23.8 |

Supplementary Table S3. Degummed silk fibers with single turned slip knot: Strength, average friction stress/strength over 0% - 40% of the strain at break, toughness modulus (T_k_), toughness modulus after knot unfastening of knotted fibers (T_u_’).

| Material | Knot topology | Sample number | Strength [MPa] | Friction stress/strength | Toughness modulus, T_k_ [J/g] | Toughness modulus after unfastening, T_u_' [J/g] |
| --- | --- | --- | --- | --- | --- | --- |
| Degummed silk | STSK | 1 | 396 | 13.1% | 27.3 | 8.4 |
| Degummed silk | STSK | 2 | 508 | 4.9% | 48.0 | 34.4 |
| Degummed silk | STSK | 3 | - | - | - | - |
| Degummed silk | STSK | 4 | 325 | 1.4% | 22.8 | 21.6 |
| Degummed silk | STSK | 5 | 675 | 0.6% | 67.1 | 64.4 |
| Degummed silk | STSK | 6 | 452 | 3.0% | 24.4 | 16.0 |
| Degummed silk | STSK | 7 | 410 | 9.0% | 36.8 | 19.5 |
| Degummed silk | STSK | 8 | 401 | 6.0% | 25.3 | 17.2 |
| Degummed silk | STSK | 9 | - | - | - | - |
| Degummed silk | STSK | 10 | - | - | - | - |
| Degummed silk | STSK | 11 | 420 | 7.5% | 30.3 | 17.0 |
| Degummed silk | STSK | 12 | 223 | 8.2% | 18.8 | 5.8 |

Supplementary Table S4. Degummed silk fibers with double turned slip knot: Strength, average friction stress/strength over 0% - 40% of the strain at break, toughness modulus (T_k_), toughness modulus after knot unfastening of knotted fibers (T_u_’).

| Material | Knot topology | Sample number | Strength [MPa] | Friction stress/strength | Toughness modulus, T_k_ [J/g] | Toughness modulus after unfastening, T_u_' [J/g] |
| --- | --- | --- | --- | --- | --- | --- |
| Degummed silk | DTSK | 1 | - | - | - | - |
| Degummed silk | DTSK | 2 | - | - | - | - |
| Degummed silk | DTSK | 3 | 225 | 0.5% | 5.0 | 3.3 |
| Degummed silk | DTSK | 4 | 318 | 0.7% | 15.5 | 10.8 |
| Degummed silk | DTSK | 5 | 518 | 0.0% | - | - |
| Degummed silk | DTSK | 6 | - | - | - | - |
| Degummed silk | DTSK | 7 | - | - | - | - |
| Degummed silk | DTSK | 8 | - | - | - | - |
| Degummed silk | DTSK | 9 | - | - | - | - |
| Degummed silk | DTSK | 10 | 624 | 0.0% | 32.6 | 31.0 |
| Degummed silk | DTSK | 11 | - | - | - | - |
| Degummed silk | DTSK | 12 | - | - | - | - |
| Degummed silk | DTSK | 13 | - | - | - | - |
| Degummed silk | DTSK | 14 | 455 | 2.2% | 34.4 | 32.0 |
| Degummed silk | DTSK | 15 | - | - | - | - |
| Degummed silk | DTSK | 16 | 243 | 0.4% | 7.2 | 4.9 |
| Degummed silk | DTSK | 17 | - | - | - | - |
| Degummed silk | DTSK | 18 | 370 | 0.6% | 13.6 | 13.2 |
| Degummed silk | DTSK | 19 | - | - | - | - |
| Degummed silk | DTSK | 20 | 489 | 0.4% | 35.1 | 34.1 |
| Degummed silk | DTSK | 21 | 497 | 0.2% | 57.1 | 56.3 |
| Degummed silk | DTSK | 22 | - | - | - | - |
| Degummed silk | DTSK | 23 | 391 | 0.3% | 22.2 | 21.1 |
| Degummed silk | DTSK | 24 | - | - | - | - |
| Degummed silk | DTSK | 25 | - | - | - | - |
| Degummed silk | DTSK | 26 | - | - | - | - |
| Degummed silk | DTSK | 27 | 470 | 0.8% | 45.1 | 42.9 |
| Degummed silk | DTSK | 28 | 696 | 1.2% | 47.0 | 42.8 |

Finally, some reference values of toughness modulus or strength of control raw or degummed single silk unknotted fibers (Supplementary Figures S1 and S2) are provided in Supplementary Tables S5 and S6, respectively. The average of the reported values were included in Table 1 in the main text.

Supplementary Table S5. Strength and toughness modulus of control raw silk unknotted fibers.

| Material | Sample number | Diameter [µm] | Strength [MPa] | Toughness modulus, T_u_ [J/g] |
| --- | --- | --- | --- | --- |
| Raw silk | 1 | 21 | 271 | 32.6 |
| Raw silk | 2 | 20 | 251 | 19.6 |
| Raw silk | 3 | 21 | 179 | 8.5 |
| Raw silk | 4 | 19 | 315 | 36.2 |
| Raw silk | 5 | 22 | 236 | 27.1 |
| Raw silk | 6 | 25 | 171 | 18.1 |
| Raw silk | 7 | 18 | 231 | 18.1 |
| Raw silk | 8 | 19 | 97 | 4.1 |
|  | Mean | 21 | 219 | 20.5 |
|  | St. Dev. | 2 | 68 | 11.1 |

Supplementary Table S6. Strength and toughness modulus of control degummed silk unknotted fibers.

| Material | Sample number | Diameter [µm] | Strength [MPa] | Toughness modulus, T_u_ [J/g] |
| --- | --- | --- | --- | --- |
| Degummed silk | 1 | 12 | 339 | 15.0 |
| Degummed silk | 2 | 8 | 646 | 43.5 |
| Degummed silk | 3 | 12 | 441 | 47.9 |
| Degummed silk | 4 | 12 | 415 | 17.6 |
| Degummed silk | 5 | 12 | 544 | 26.4 |
| Degummed silk | 6 | 14 | 490 | 24.8 |
| Degummed silk | 7 | 14 | 496 | 18.7 |
| Degummed silk | 8 | 14 | 474 | 40.1 |
|  | Mean | 12 | 481 | 29.3 |
|  | St. Dev. | 2 | 91 | 12.8 |


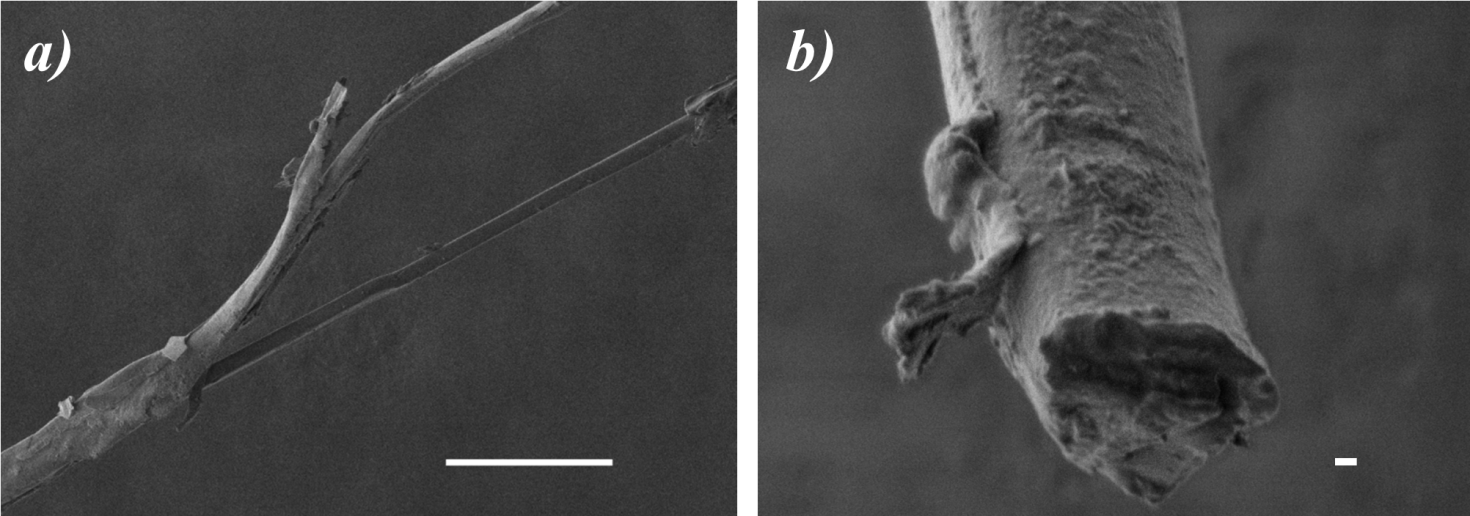


Figure S1: a) SEM image of a natural silk fiber (bave), where the seracin coating, which binds two core brins (detailed in b), scale bar: 1 µm), is broken (scale bar: 100 µm).


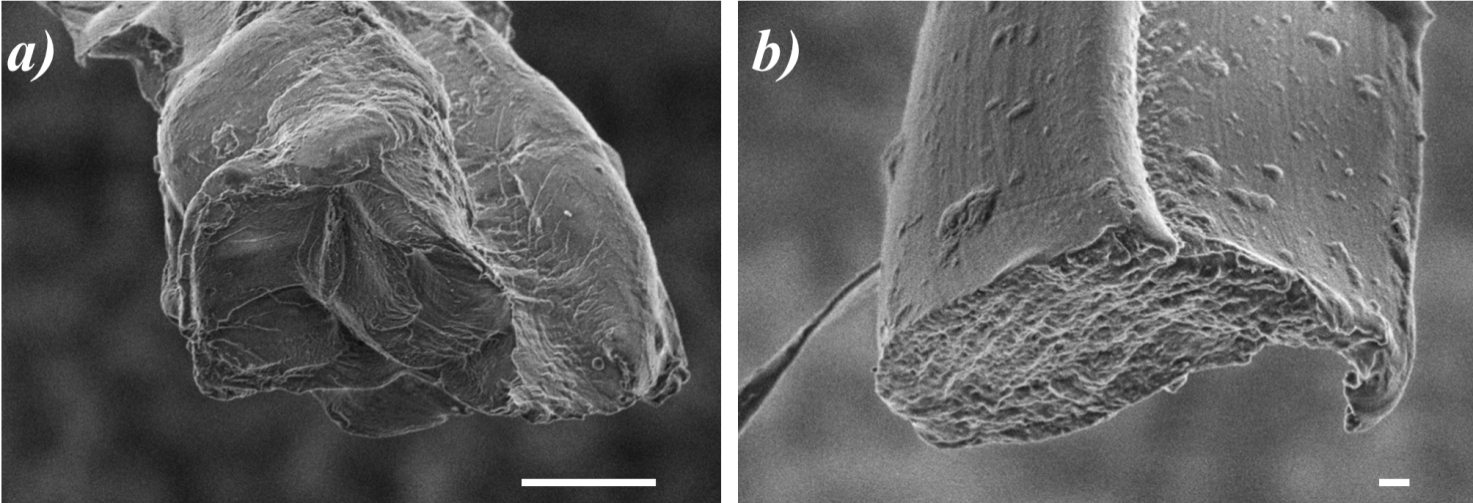


Figure S2: a) Cross-section of a natural silk fiber, consisting of two fibroin cores coated by a seracin layer (scale bar: 10 µm); b) Cross section of a degummed silk fiber (scale bar: 1 µm).

**References**

[S1] Ashby MF, Elsevier 2011, Materials Selection in Mechanical Design Butterworth-Heinemann, Burlington, MA, USA.
